# Forensic Analysis of Venezuelan Elections during the Chávez Presidency

**DOI:** 10.1371/journal.pone.0100884

**Published:** 2014-06-27

**Authors:** Raúl Jiménez, Manuel Hidalgo

**Affiliations:** 1 Department of Statistics, Universidad Carlos III de Madrid, Getafe, Madrid, Spain; 2 Department of Social Sciences, Universidad Carlos III de Madrid, Getafe, Madrid, Spain; Universita' del Piemonte Orientale, Italy

## Abstract

Hugo Chávez dominated the Venezuelan electoral landscape since his first presidential victory in 1998 until his death in 2013. Nobody doubts that he always received considerable voter support in the numerous elections held during his mandate. However, the integrity of the electoral system has come into question since the 2004 Presidential Recall Referendum. From then on, different sectors of society have systematically alleged electoral irregularities or biases in favor of the incumbent party. We have carried out a thorough forensic analysis of the national-level Venezuelan electoral processes held during the 1998–2012 period to assess these complaints. The second-digit Benford's law and two statistical models of vote distributions, recently introduced in the literature, are reviewed and used in our case study. In addition, we discuss a new method to detect irregular variations in the electoral roll. The outputs obtained from these election forensic tools are examined taking into account the substantive context of the elections and referenda under study. Thus, we reach two main conclusions. Firstly, all the tools uncover anomalous statistical patterns, which are consistent with election fraud from 2004 onwards. Although our results are not a concluding proof of fraud, they signal the Recall Referendum as a turning point in the integrity of the Venezuelan elections. Secondly, our analysis calls into question the reliability of the electoral register since 2004. In particular, we found irregular variations in the electoral roll that were decisive in winning the 50% majority in the 2004 Referendum and in the 2012 Presidential Elections.

## Introduction

Hugo Chávez was elected President of Venezuela in 1998 and ruled the country until his death in 2013. He won four consecutive presidential elections (1998, 2000, 2006 and 2012) and a recall referendum (2004), convened against him by opposition forces. He also proposed several major reforms that were approved in national referenda (two held in 1999, one in 2000 and another in 2009). In addition, his party won an overall majority in the National Assembly in three parliamentary elections that took place during his presidency (2000, 2005 and 2010), and in all regional and local elections. His sole election defeat came in the 2007 constitutional referendum, when he attempted a radical socio-political reform. This electoral record could be overshadowed, however, by the allegations of fraud made by opposition sectors since the 2004 Recall Referendum [1].

The electoral law, approved in Venezuela in 1997, established the automation of the vote count. In the period between 1998 and 2000, the vote count was carried out both manually and automatically. However, since 2004 the results come exclusively from a computer center, where the data from the voting machines distributed throughout the country are centralized. Another important characteristic that differentiates the electoral processes before and after 2004 is the composition of the governing body of the elections, the National Electoral Council (CNE in Spanish). The National Assembly, which was controlled by the ruling coalition, appointed an openly pro-government management body. Four out of the five current CNE's rectors lean strongly towards the ruling party and only one to the opposition forces. Although the CNE has improved the transparency and reliability of the electoral system, particularly since 2006, the fact is that the Venezuelan electoral authority has taken controversial decisions that have only ever favored the government and never the opposition [2].

Despite the frequent use of the term, there is ambiguity regarding what is and what is not electoral fraud. What may constitute fraud in one country, or at a particular moment, may not be considered as such in another. Nonetheless, any irregular action that is performed with the intention of altering the development of an election or election-related materials, with the aim of affecting its results, may be considered a fraud [3]. In Venezuela, allegations of fraud are not new, but they have become more frequent since 2004. Apart from allegations of manipulations of the vote count, the opposition has made other claims, including manipulation of the electoral register, coercion of public servants and the electorate, and misuse of public resources and funds for electioneering. There have also been some accusations of polling station violations and the destruction of electoral material. A summary of the alleged electoral irregularities under *Chavismo* can be found in http://www.americasquarterly.org/electoral-irregularities-under-chavismo-tally. Links to several dozens of documents about them are available at http://esdata.info and http://www.sumate.org.

Some electoral irregularities may leave traces in the form of numerical anomalies. If this is the case, they can be detected by appropriate statistical methods. The main idea underlying these methods is the comparison between observed values of statistics based on the vote count and their expected values. When we say expected value, we usually mean the regular value in a free and fair election. Therefore, large discrepancies between observed values and expected ones (*outliers*) are usually interpreted as statistical evidence regarding the fairness of an election. Benford's test [4] and many other tools used in election forensics [5] are examples of these methods. The application of statistical mechanics concepts has helped notably in the understanding of statistical regularities in the vote count [6]–[8], providing new insights for the forensic analysis of elections [9]. But the mere presence of outliers is not a proof of fraud, even less of an *outcome-determinative* fraud, “where the fraud affects the outcome of the election such that the winners and losers are different from what they would have been had the fraud not be committed” [3]. Elections are complex processes where errors and unforeseen events frequently occur. Some of them may even constitute serious irregularities and may generate outliers but may not, however, affect aggregate results. Nevertheless, the presence of electoral irregularities that systematically favor one electoral option is another issue. The political implications may be serious when the overall results are affected. For this reason, we are not only interested in detecting outliers that may be the trace of a fraud, but also in evaluating if they are correlated with a bias in the vote count and if this could have been a determining factor in Chávez's electoral victories.

This paper proceeds as follows. In the next section we describe the election data under study. Then, we apply a battery of election fraud forensic tests, which provide consistent and complementary results. Thereafter, we turn to a discussion on the integrity of Venezuelan elections and present some final conclusions.

## Data Description

In our study we considered the following Venezuelan elections:

- Presidential elections 1998, 2000, 2006 and 2012
- Referenda 1999, 2004, 2007, and 2009
- Parliamentary elections 2005 and 2010

Therefore, we took into account every year of national-level elections since Chávez first won the presidency of Venezuela until his death. However, for the 2000 general elections, known as ‘Mega-elections’ because every single official was re-elected, we only considered data from the presidential elections. In 1999 there were two referenda, one in April and one in December, and one election in July for the seats of the National Constituent Assembly (NCA). During the April referendum, two queries were made: about the convening of the NCA to draft a new constitution and about the approval of the basis for this constituent process. In December, the new constitution was adopted by national referendum. We only considered the April referendum due to the lack of available data for the July elections and the December referendum at the level of breakdown we require for our analysis. The official data (available at http://www.cne.gov.ve/web/index.php) has been downloaded and stored in spreadsheets in http://esdata.info/, where the reader can also find additional information on each election.

For our analysis, we have taken into account data at the least aggregation level. The polling cluster that collects this data has been denominated differently in diverse elections: voting table, electoral notebook, voting machine, etc. To avoid confusion, we will refer to it as *electoral unit* [10]. For all the presidential elections and referenda, a small number of electoral units outside of the country were excluded. We did this to standardize the data set. On the one hand, these units were peculiar and negligible for total results. On the other hand, there were no electoral units abroad in parliamentary elections. We also excluded a very small number of electoral units with missing data or without valid votes that could arise from technical problems. Thus, the average of registered voters by electoral unit is very similar in the data set under study. Roughly this figure comes down to 500, except for the 2000 Presidential Elections, which is 1126. However, the number of electoral units almost doubled between 1998 and 2012, from 20,026 units to 38,853, showing a strong growth in voter registration.

Unlike in an earlier version of this paper [11], where we analyzed only some of the elections under consideration, we do not distinguish between data coming from automated polling stations or not. But we look at the same variables per electoral unit. Namely:

1. Number of votes for Chávez. This means, votes for him in presidential elections, for his proposals (in referenda), and for the endorsed candidates by the ruling party (in parliamentary elections)
2. Number of valid votes
3. Number of registered voters
4. Polling center to which the electoral unit belongs

For each election, we consolidated these data in one set, labeled with the year of the election, except for the 1999 and 2007 referenda and the 2010 Parliamentary elections, for which there are two data sets. 1999a, 1999b, 2007a and 2007b are the abbreviations to refer to the data associated to the two questions considered in the referenda of 1999 and 2007. The 2010 Parliamentary elections were preceded by an electoral reform. Under the approved system, 70% of the 165 deputies of the National Assembly were elected on a first-past-the post system and 30% on a party list. The results are considered in two separate sets, labeled 2010a and 2010b, respectively. Each polling center is identified by a code. The numbers were re-labeled. We used the old labels for elections and referenda previous to 2005 and the new ones for elections and referenda from 2005 onwards. The conversion table and the election data under consideration are available at http://elecionforensincs.com.es/. Table 1 shows the percentages of votes for Chávez and the voter turnout of the elections under study.

**Table 1:** Percentage of votes favoring Chávez and voting turnout.

Election	1998	1999a	1999b	2000	2004	2005	2006	2007a	2007b	2009	2010a	2010b	2012
[%] Chávez	56.20	87.75	81.74	59.76	59.09	85.50	62.84	49.29	48.94	54.85	48.20	48.72	55.07
[%] Turnout	63.45	37.65	37.65	56.63	69,92	25.26	74.69	55.90	55.90	69.92	66.45	66.45	80.56

## Data Analysis

### Second-Digit Benford's Law and Venezuelan elections

The Benford test for the second significant digit is one of the most commonly-used tools in election forensics. It has been previously used to analyze the 2000 Presidential Elections and the 2004 Recall Referendum [10]. Unlike this analysis, we do not distinguish between data coming from automated electoral units or otherwise. The heuristics behind the test can be summarized in our context as follows:

From polling places that collect election data with 10 or more votes favoring Chávez, consider the proportion 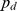 to be those having a number of votes favoring Chávez in which a second significant digit equals *d*. The restriction of 10 or more votes is required only for the existence of the second significant digit. If the election is fair, the frequency distribution 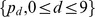 must fit Second-Digit Benford's law
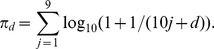



Discrepancies between the frequency distribution and the law may be interpreted as evidence of fraud of various kinds.

The most accepted discrepancy measure between the frequencies distribution and the law is the Pearson's chi-square statistic
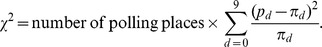



The statistics is the basis of an uncritical practice to test the null hypothesis *H_0_*: The data is consistent with the Benford's law for the second significant digit. If the chi-squared *p*-value  =  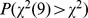 is small (less than 0.05, for example) it is assumed that there is an evidence against *H_0_*. Here and elsewhere, 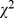 denotes a chi-squared random variable with 9 degrees of freedom. Overall, the usefulness of a chi-squared *p*-value is drastically affected by the sample size (number of polling places, in our case). For a correction of *p*-values, Pericchi and Torres [10] propose the *Bayesian posterior probability with Uniform priors*. This measure, denoted by *P*(*H_0_* |data), can be understood as the probability of being right when we assert the hypothesis *H_0_* is true.

Despite its widespread use, the application of Benford's test has been severely criticized [12]. It is shown that deviations from the law can occur when elections are fair (*false positives*) and, conversely, that they might not occur in fraudulent elections (*false negatives*). Mebane [13] has responded to these criticisms, arguing that there was an improper application of the law. He is careful to point out that the right implementation of the test depends on the data aggregation level chosen. We now proceed to analyze three different aggregation levels.

**Figure 1:**
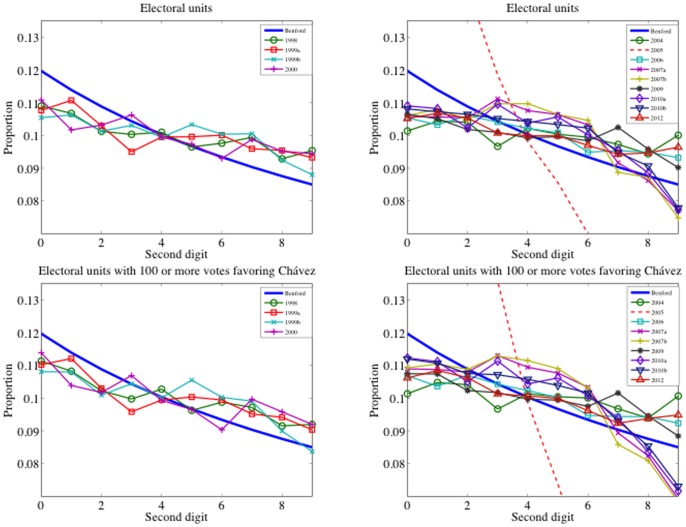
Second-Digit Benford's law and proportions on electoral units. Top panels: Electoral units with 10 or more votes for Chávez. Bottom panels: Electoral units with 100 or more votes for Chávez. Left panels: Presidential elections and referenda previous to 2004. Right panels: Elections and referenda between 2004 and 2012. The proportions of the 2005 Parliamentary Elections are partially out of the *y*-axis range.

Firstly, let us consider the electoral units with 10 or more votes favoring Chávez. Fig. 1 (top panels) shows the shapes of the frequency distributions for each Venezuelan election. Although at first glance there is no election that matches the law, the distributions of some elections held after 2004 are further away from it, particularly the 2005 Parliamentary Elections. There is consensus that some factors other than fraud may affect the distribution of the second digit [14]. This could have been the case of the 2005 elections, which represents the extreme outlier of our case studies. These elections were boycotted by the opposition, which called for abstention and withdrew from the elections at the last minute, claiming that the secrecy of the ballot was not guaranteed. Chávez's allies would have therefore won easily, with or without fraud. Thus, there is no reason to believe that any fraud existed. But neither can we rule out that the boycott affected the distribution of the second digit. Certainly, it had a considerable impact on the voter turnout, which was extremely low (25.26%). The chi-squared statistics are consistent with our previous observation from the shapes of the distributions. The observed value is much larger between 2004 and 2012 than before 2004, and is extremely large for 2005 (Table 2). However, both the *p*-values and the Bayesian posterior probabilities proposed by Pericchi and Torres are negligible at this aggregation level, except for the 2000 elections.

**Table 2:** Benford's test statistics based on electoral units with 10 or more votes for Chávez.

Election	1998	1999a	1999b	2000	2004	2005	2006	2007a	2007b	2009	2010a	2010b	2012
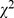	95.17	94.16	101.1	49.47	203.93	1977.0	151.74	176.88	195.77	197.68	157.25	143.33	192.58
*p*-value	0.000	0.000	0.000	0.000	0.000	0.000	0.000	0.000	0.000	0.000	0.000	0.000	0.000
*P*(*H_0_*|data)	0.000	0.000	0.000	0.9993	0.000	0.000	0.000	0.000	0.000	0.000	0.000	0.000	0.000

Statistical tests examining the fit of Benford's law have more power on data with several significant digits. In fact, in accounting fraud detection, among other fields, it is a usual practice to restrict the analysis to data with three or more significant digits [15]. But to our knowledge, this restriction has not been used previously in election forensics. We now examine electoral units with 100 or more votes favoring Chávez, that is, with three significant digits. We note that, under this restriction, the distribution of the number of votes favoring Chávez has positive skewness for all the elections that we are considering. This is a property that will satisfy a Benford dataset to prevent false positives [16]. Without the restriction, the skewness is negative for many of the elections under study. Also, it is important to note that the restriction only excludes a set of electoral units that does not significantly change the overall results. In any election, at least 83% of the electoral units have at least 100 voters for Chávez, except the 2005 Parliamentary Elections where only 59% of the units had this property. Fig. 1 (bottom panels) shows also the shapes of the frequency distributions for the restricted data set. Although there is not an evident difference with the previous plots, the Pearson's chi-square statistic is notably lower in many of the elections, remaining larger between 2004 and 2012 than before 2004. Interestingly, while the *p*-values are still almost zero, the Bayesian posterior probabilities change abruptly when we consider only electoral units with 100 or more votes favoring Chávez. According to this measure, we should reject *H_0_* exclusively from 2004 onwards (see Table 3). The restriction appears to be useful in preventing false positives for analyses based on the Pericchi-Torres method. The 1998 Presidential Elections provide an excellent example. The elections were legitimized both by international observers and by political parties and have been presented as an example of a fair election [17]. The Bayesian posterior probability obtained from this election switches from almost 1 to almost 0 whether we consider the restriction or not. Something similar occurs with the 1999 referenda, which are not questioned.

**Table 3:** Benford's test statistics based on electoral units with 100 or more votes for Chávez.

Election	1998	1999a	1999b	2000	2004	2005	2006	2007a	2007b	2009	2010a	2010b	2012
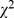	53.04	56.57	69.19	35.26	189.78	55178.	114.97	203.76	232.33	135.08	157.89	122.13	132.68
*p*-value	0.000	0.000	0.000	0.001	0.000	0.000	0.000	0.000	0.000	0.000	0.000	0.000	0.000
*P*(*H_0_*|data)	1.000	0.997	0.388	1.000	0.000	0.000	0.000	0.000	0.000	0.000	0.000	0.000	0.000

**Figure 2:**
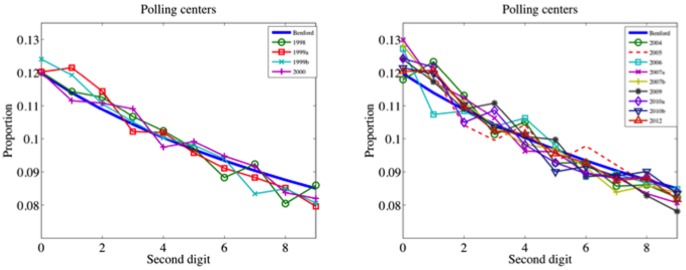
Second-Digit Benford's law and proportions on polling centers. Left panel: Presidential elections and referenda previous to 2004. Right panel: Elections and referenda between 2004 and 2012.

It is well known that Benford's test can be applied to data that are distributed across multiple orders of magnitude. The votes per electoral unit are certainly not. They are less than 600 in almost any Venezuelan election. The bound is (twice) larger only in 2000. The natural way to span these data to higher orders of magnitude is to consider outcomes per polling center. Polling centers may combine multiple electoral units in the same voting place with a number of votes for Chávez above 8000. Fig. 2 shows the observed proportions at this aggregation level, and, unlike in the two previous cases, all elections look to be close to the law. The observed 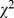 statistics are significantly smaller. But, once again, we observe larger values from 2004 onwards, with *p*-values less than 0.1 in 2004, 2006, 2007a, 2009 and 2010a (see Table 4). It comes as no surprise that the Bayesian posterior probabilities based on polling centers are almost 1 for all the elections. Pericchi and Torres have already reported that their measure overestimates the fit in cases where the data is aggregated. We agree with Pericchi and Torres [10] that the implementation of the method must be based on data at the lowest level of aggregation, mainly because aggregated data may mask some kind of data manipulations made at the lower aggregation level. But also, we must take into account that the power of the test relies on the order of magnitude of the data. Finding a trade-off between data at low aggregation level and data with several significant digits from elections does not seem to be an easy problem. The analysis per electoral units with 100 or more votes for Chávez could be an equilibrium point for the Venezuelan election data.

**Table 4:** Benford's test statistics based on polling centers.

Election	1998	1999a	1999b	2000	2004	2005	2006	2007a	2007b	2009	2010a	2010b	2012
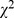	9.27	10.99	10.34	6.20	14.90	11.72	16.15	16.95	12.09	20.52	18.08	12.02	9.86
*p*-value	.4127	.2764	.3237	.7197	.0937	.2296	.0638	.0495	.2083	.0150	.0343	.2122	.3619
*P*(*H_0_*|data)	1.000	1.000	1.000	1.000	1.000	1.000	1.000	1.000	1.000	1.000	1.000	1.000	1.000

Beyond the controversies concerning the application of Second-Digit Benford's law for fraud detection, we can extract at least one conclusion from our analyses: 2004 appears to be an inflection point in which the Venezuelan elections begin to move away from the law. A recent study based on authentic and synthetic election data reports that the non compliance of the law is associated with fraud at least in 50% of cases [18]. With the exception of the 2005 elections, we cannot provide an explanation of why the law fails for the referenda and elections held between 2004 and 2012.

### Venezuelan election fingerprints

**Figure 3:**
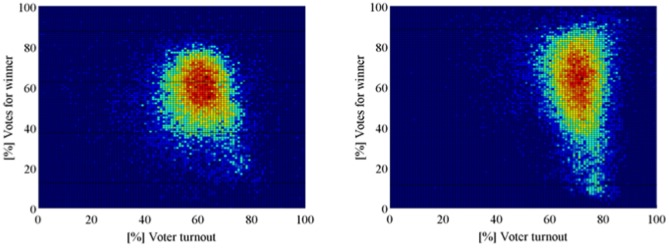
Election fingerprints: 3D-histograms of the number of electoral units for a given voter turnout (*x*-axis) and the percentage of votes for Chávez (*y*-axis). Left panel: 1998 Presidential elections. Right panel: 2004 Recall Referendum. Color represents the number of electoral units with corresponding (*x,y*)-coordinates.

The second-digit Benford's law and other tests based on the frequency of digits [18], [19] are useful election forensics tools, especially if we ignore the substantive context of the election under study [14]. A different category is based on the analysis of number of votes, turnout, and other electoral variables [5], [9], [20], [21]. These analyses have already been used to examine the Venezuelan referenda of 2004 [21], 2007 and 2009 [20]. One of the advantages of these methods is the easier interpretation of their outputs in terms of electoral behavior. Within this category, the work of Klimek et al. [9] has attracted special attention [22], [23]. In their paper, the authors show new evidence of election fraud in Russia and Uganda, and discuss several types of results, including: different characterizations of the probability distribution of votes and models for the joint distribution of the percentage of votes for winner and voter turnout. Although these issues have been investigated in seminal works [5], Klimek et al. introduce several novelties. One of them is a suitable data representation that they call the *election fingerprint*. Their fingerprints are the 3D-histograms of the number of electoral units for a given voter turnout and the percentage of votes for the winner (for Chávez in our case). From these figures they make inference on possible processes and mechanisms that lead to the overall election results. We show the election fingerprints of the presidential elections 1998 and the recall referendum 2004 (Fig. 3).

According to Klimek et al., fit models for fingerprints of fair elections should correspond to bivariate Gaussian distributions. They test this hypothesis with many countries; including Austria, the Czech Republic, Finland, France, Poland, Romania, Spain, and Switzerland. They also consider non-fraudulent mechanisms that can explain discrepancies from the bivariate Gaussian distribution, e.g. the heterogeneity of the Canadian population. In addition, they discuss fraudulent processes that may contribute to deviations from their fair election model, such as ballot stuffing and coercion to obtain complete turnout and votes for winner. The 1998 Venezuelan Presidential Elections are very close to their model of fair elections, while the 2004 Recall Referendum is farther from it. Leaving aside whether or not there was fraud in 2004, these two electoral processes provide two different fingerprint models for the same electoral population, corresponding to two crucial moments. We are interested in classifying the elections according to the election fingerprint model that better fits the data. For that, we rehearsed with several classification methods, obtaining similar results. Below, we show the outputs of a quadratic classifier that fits multivariate normal densities with covariance estimates stratified by group (1998 and 2004). We selected this method because it relies on the Gaussianity hypothesis of Klimek et al. The classifier provides a simple rule to determine when an electoral unit is an observation that most likely corresponds to the 1998 model rather than to the 2004 model. The results allow for the elections to be grouped into four categories, according to the shape of their fingerprints and the percentage of electoral units classified into the 1998 model, which we will denote by [%] Mod.98.

**Figure 4:**
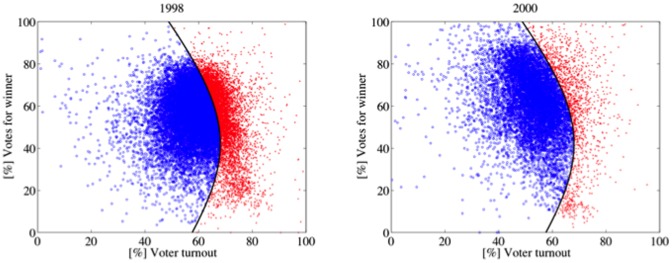
Gaussian quadratic classifier: The black line represents the decision boundary. Each electoral unit represented by a blue circle has been classified as an observation of the Gaussian fit model based on 1998 data. Otherwise, it is represented by one red x. In both elections, the units are clustered around their respective averages of turnout and votes for Chávez. By excluding some units with turnout between 60% and 80%, and low support for Chávez (less than 20%), the scatterplots appear to be normally distributed.

**Figure 5:**
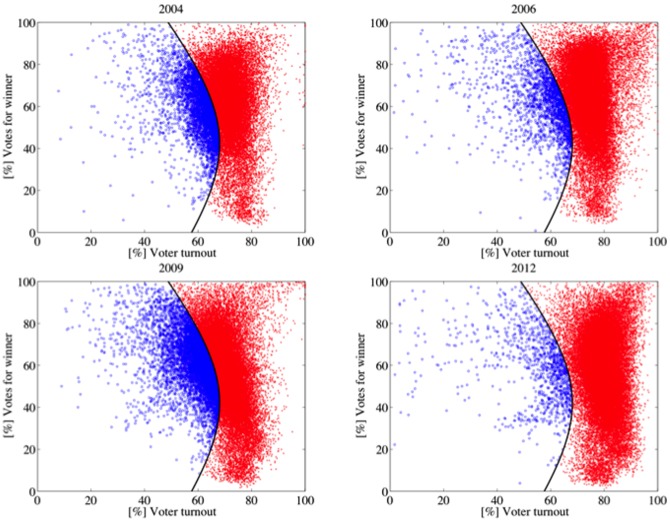
A high percentage of electoral units from the 2006 and 2012 elections and the 2004 and 2009 referenda cannot be classified as observations of the Gaussian fit model based on 1998 data. The scatterplots have many units with high turnout and high support for Chávez.

**Figure 6:**
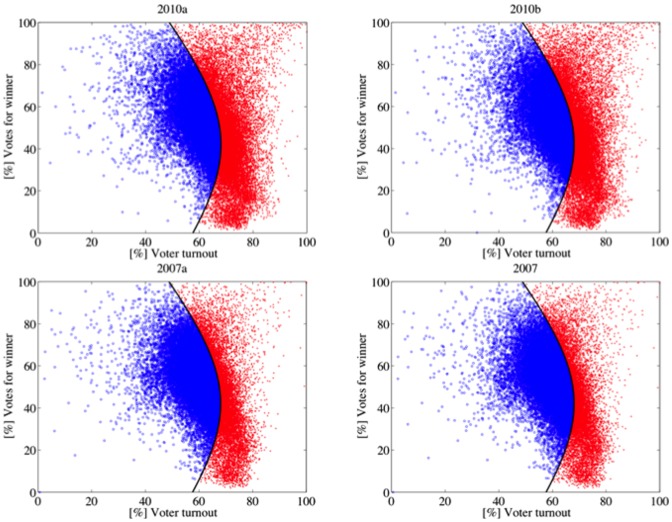
The scatterplot of the 2007 referendum and the 2010 parliamentary elections has a shape similar to the 2004 case. However, the set of electoral units close to the top right corner is less dense (2010) or negligible (2007). Additionally, their [%] Mod.98 values are considerably high, as well as the percentage of electoral units classified into the 2004 model. These elections seem to fit a true mixture model.

**Figure 7:**
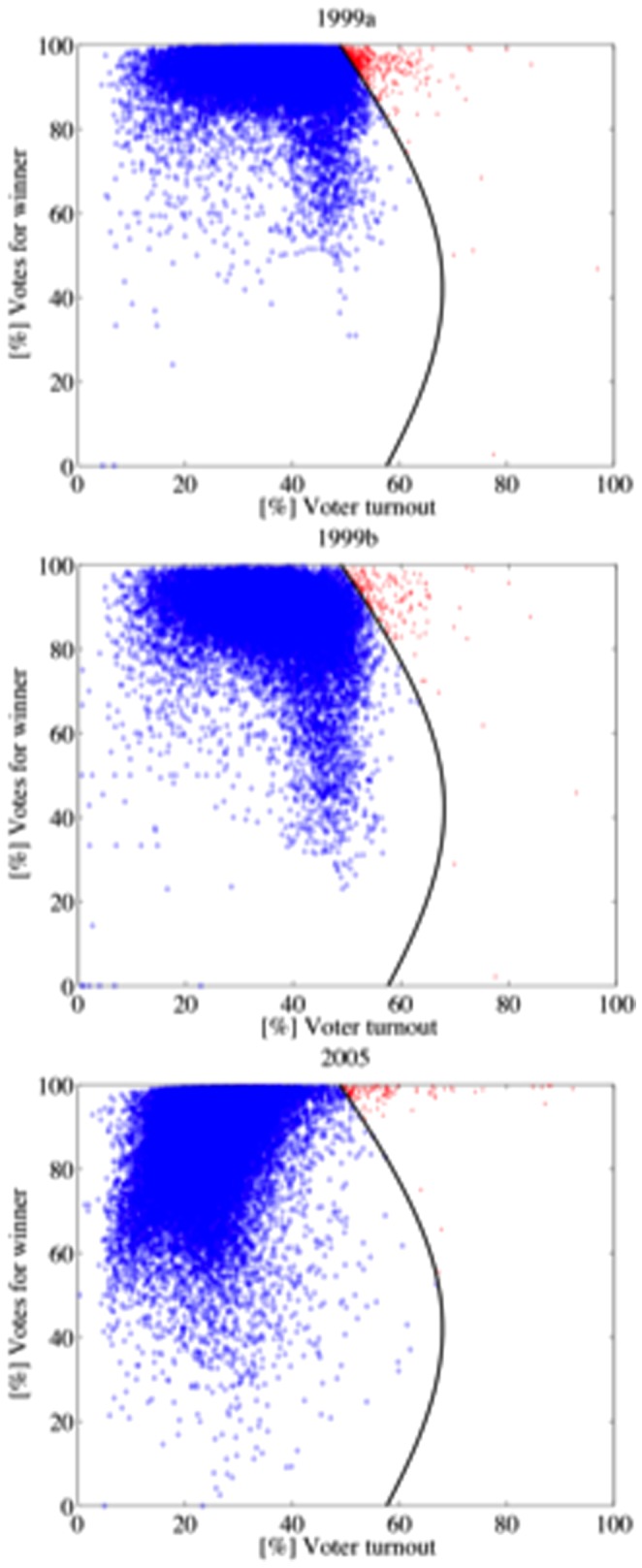
The scatter plots of the 1999 referenda and the 2005 elections are mainly characterized by the low voter turnout and high votes for Chávez (**Figure 6**). This shape is a consequence of the low opposition turnout in 1999 and its almost total absence in 2005.

Table 5 shows the [%] Mod.98 values of every election. The elections in the first category (1998 and 2000) have high [%] Mod.98 values and show a similar shape (see Fig. 4). Their electoral units are, roughly, normally distributed around their respective averages of turnout and votes for Chávez. Elections and referenda of the second category (2004, 2006, 2009 and 2012) have low [%] Mod.98 values, in particular 2006 and 2012. They share a similar shape, different from the above (see Fig. 5). These elections and referenda have many units with high turnout and high support for Chávez. According to Klimek et al., electoral units of this type may be associated with *incremental and/or extreme fraud*. Incremental fraud means that ballots for one candidate are added or votes for other candidates are taken away. Extreme fraud corresponds to reporting a complete turnout and almost all votes for a single candidate. The 2007 Constitutional Referendum and the 2010 Parliamentary Elections deserve a special category. Although their shapes are very similar to the second category (Fig. 6), the set of electoral units close to the top right corner of the figure is less dense (2010) or negligible (2007). Additionally, their [%] Mod.98 values are considerably high, as well as the percentage of electoral units classified in the 2004 model. These elections seem to fit a true mixture model. The last category (1999 referenda and 2005 elections) is mainly characterized by the low voter turnout and high votes for Chávez (Fig. 7), consequence of the low opposition turnout (1999), even of its almost total absence (2005). The [%] Mod.98 values of this category are extremely high.

**Table 5:** Percentage of electoral units classified as observations from the election fingerprint model of 1998.

Election	1998	1999a	1999b	2000	2004	2005	2006	2007a	2007b	2009	2010a	2010b	2012
[%] Mod.98	72.03	98.21	99.05	86.82	18.25	99.50	06.69	69.30	69.54	23.14	54.38	56.22	02.83

**Figure 8:**
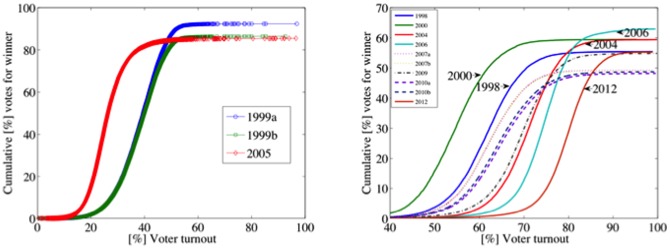
Cumulative number of votes favoring Chávez as a function of turnout. The shape of every referendum/election is a sigmoid that reaches a plateau at the maximal vote count for Chávez. The curves of 2004, 2006, and 2012 increase close to complete turnout.

A simple way to summarize the outputs that we have discussed is by plotting the cumulative number of voters favoring the winner as a function of the turnout [9]. This curve is obtained by computing, for each turnout level, the percentage of votes favoring Chávez from units with this level or lower. What we expect, if the election is fair, is a sigmoid that reaches a plateau at the maximal vote count for Chávez, at turnout levels of much less than 100%. The referendum of 1999 and the 2005 elections develop this shape, reaching the plateau to a high support for Chávez at low levels of voter turnout. These curves are particular, a consequence of the low turnout in these elections that we have already commented on. They are plotted separately (Fig. 8, left panel). The 1998 and 2000 presidential elections develop the expected shape at moderate levels of turnout (Fig. 8, right panel). In contrast to these elections, the curves of 2004, 2006, and 2012 increase to close to 100% turnout. In a middle range, we can locate the rest of the curves (2007, 2009 and 2010). Although the 2009 curve is very close to that of 2004, it does not increase at large turnout values.

The analysis carried out suggests that 2004 is a breakpoint in the voting behavior of the Venezuelans. The election fingerprints of the presidential elections previous to 2004 fit well the model of fair elections proposed by Klimek et al. The low opposition turnout in the 1999 referendum and parliamentary elections 2005 can explain the deviations of these processes from the Gaussian model. The recall referendum showed a new Venezuelan election fingerprint, that was farther from the Gaussian fair election model. Its shape is shared by the referenda and elections held between 2004 and 2012, in particular by the 2006 and 2012 presidential elections and the 2009 referendum, processes that were characterized by many electoral units with high voter turnout and strong support for Chávez. Many factors can explain the presence of units with these characteristics. Certainly, as Klimek et al. argue, one may be ballot stuffing and/or coercion in some electoral units. But there are also other non-fraudulent devices that can explain these results. As Mebane concludes [22], some electoral districts may be “special places”. In them, a high percentage of registered voters can vote for the same option, and this does not necessarily indicate the presence of any type of fraud. The idiosyncrasy of each electoral area has to be looked at. Venezuela is a polarized country, where there are many highly politicized areas. For this reason, one should expect electoral units with high level of support for Chávez or, conversely, for the opposition [24]. What we discuss next is the detection of atypical support in electoral units, relative to the support obtained in the polling center to which the unit belongs. Thus we solve the problem with the special places. Explicitly, we are interested in ascertaining if this atypical support is characteristic of pro-Chávez polling centers or not.

### Statistical detection of irregular support

As we have already discussed [21], Venezuelan voters can choose the polling center where they vote. But, in polling centers with two or more electoral units, the voters are assigned to the units according to a pseudorandom criterion. Therefore, conditioning on the results by polling centers, the number of votes per electoral unit follows a Hypergeometric distribution. Specifically:

- Denote by *V* the number of votes favoring Chávez in a given electoral unit.
- Let *p* be the proportion of votes favoring Chávez over the number of registered votes at the center to which the unit belongs.
- Denote *n* and *m* be the number of voters registered in the electoral unit and in the polling center.

Then, given *p, n*, and *m*, *V* follows a Hypergeometric distribution with expected value equals to *pn* and variance equals to 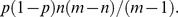 Thus, a standardized measure of regularity of the number of votes favoring Chávez in the electoral unit is the *Z*-score
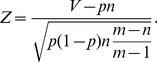



**Figure 9:**
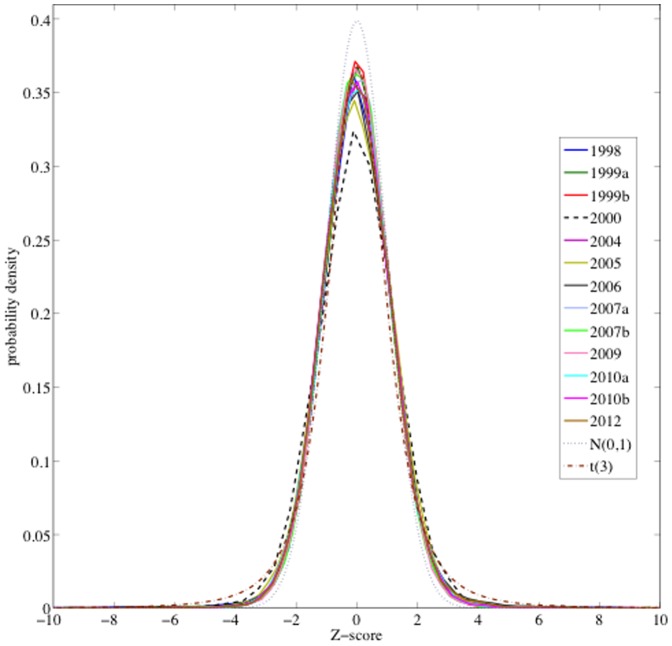
The distributions of *Z*-scores of different elections collapse on a *t*-student with 3 degrees of freedom. Only the 2000 elections show slightly heavier tails.

*Z*-scores far from zero imply irregular support in the electoral unit, no matter how “special” or “standard” is the polling center to which the unit belongs. When *n* is large, and *m* much larger than *n*, the distribution of the *Z*-score should be approximately a standard normal *N*(0,1). However, some irregularities may generate large values of *Z*, out of any normal confidence interval. Examples of these irregularities are ad hoc decisions on the final allocation of voters, taken on the election-day to solve fails on touch-screen machines. We will call *non-fraudulent irregularity* any unforeseen action that affects the vote distribution of the electoral units in a polling center without affecting significantly the vote distribution at the center. Non-fraudulent irregularities may occur with high probability due to the complexity of the electoral processes. Therefore, the distribution of the *Z*-scores should have heavier tails than the normal distribution. In fact, the *Z*-scores of the elections collapse on a *t*-student. With the possible exception of the 2000 elections, the goodness of fit is extremely good for a *t*-student with 3 degrees of freedom (Fig. 9), hereafter denoted by *t*(3). As we commented, 2000 was a mega-election, where every elected office in the country was elected. Thus, we expect more non-fraudulent irregularities in these elections than in any other and, consequently, heavier tail distribution for their *Z*-scores. But, leaving aside some minor loss of accuracy for the 2000 case, we can assume that the *Z*-scores of any election are approximately distributed according to a *t*(3). This fit will be used to simulate *Z*-scores for a bootstrap model, which is employed only for illustrating the asymptotic normality of the test statistics that we discuss below. These statistics, that we will name *standardized differences*, are based on the *Z*-scores but their asymptotic distribution does not depend on the goodness of the fit of the *t*(3)-distribution.

If an election is fair, including that election resources are distributed with equity among the polling centers, *Z*-scores farther from zero should be product of chance. This covers extreme *Z* values generated by non-fraudulent irregularities on a random set of electoral units. Hence we consider:

- The set of the *k* electoral units with *Z*-score farther from zero, which we will denote by *M_k_*.
- The null hypothesis *H_1_*: all the electoral units have the same probability to be in *M_k_*.

We propose a test for *H_1_* based on one developed for the study of the 2004 Recall Referendum [21]. It relies on the classical confidence intervals for the ratio estimator [25].

Let 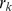 be the proportion over valid votes of votes for Chávez on *M_k_*. Denote by *R* the same proportion but computed on all the electoral units under study. Let *T_i_* be the total valid votes at the electoral unit *i*, *V_i_* the number of votes for Chávez, and
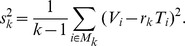



Denote by *K* the total number of electoral units, and by 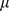 the average of valid votes per electoral unit. Now consider the estimated variance of 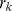 defined by
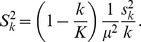



Then, if *k* is large, *K-k* is much larger than *k*, and *H_1_* is true, the standardized difference
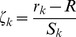
is distributed approximately as a standard normal *N*(0,1) [25]. We test *H_1_* by computing the 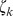 for large values of *k*. Values far away from normal confidence intervals, for a wide range of large values of *k*, are considered strong presumptions against *H_1_*. In estimating proportions, standard large sample sizes (*k*, in our case) are above 1000. We consider values of *k* between 500 and 1500, thus we are covering, from below and from above, standard large sample sizes. For all the cases, *K-k* is large enough. We also illustrate the asymptotic normality of 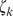 under *H_1_* from a model of fair elections based on a hierarchical bootstrap. Specifically, we generate random samples of size *K* of *Z*-scores from a *t*(3) distribution. Then we assign the *k Z*-scores farther from zero to a random sample of units. Thus, 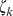 is computed from the above equations, keeping the observed values of *p*, *m*, *n* and *T_i_*, per electoral unit and polling center in each election or referendum.

**Figure 10:**
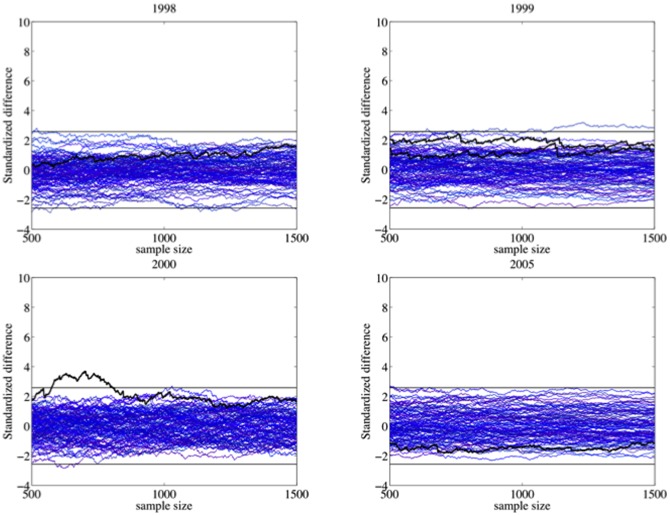
The standardized differences of the 1999 referenda and the 1998, 2000 and 2005 elections (wide black lines) are well embedded within/in the 99% normal confidence interval at large sample sizes. Standardized differences of fair elections computed from a hierarchical bootstrap model (thin blue lines) also verify the expected behavior under *H_1_*.

**Figure 11:**
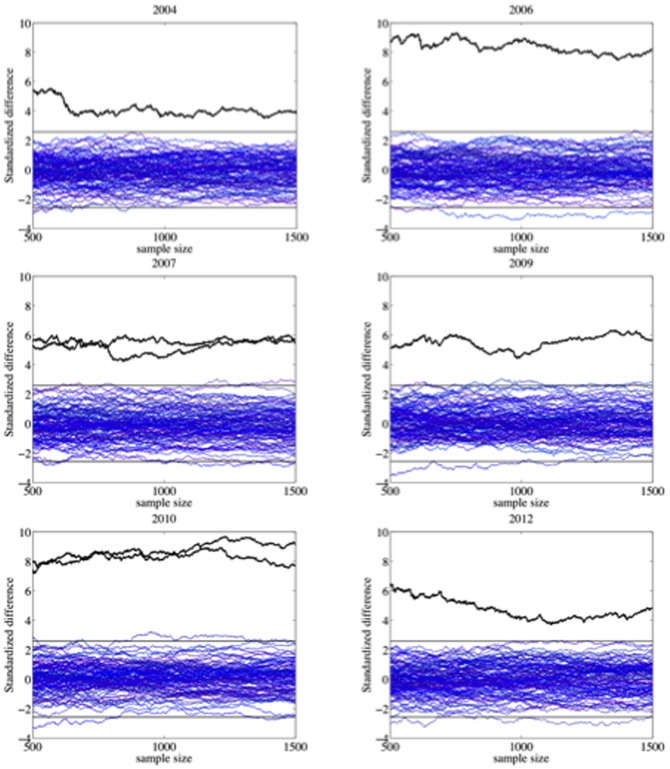
The standardized differences based on official results from 2004 onwards reach values higher than any simulation. They are well above the 99% normal confidence interval. These elections provide strong evidence against *H_1_*. The more irregular distributions of votes occurred on electoral units where the vote counting was significantly favorable to Chávez.

Figures 10 and 11 display the standardized differences computed from the official results of all the elections and referenda. For each year, we also consider the standardized differences of 100 fair elections computed from the bootstrap model discussed above. Fig.10 shows the 1999 referendum and the 1998, 2000 and 2005 elections. Fig. 11 shows the rest. We also plotted the 99% normal confidence interval (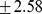) in all the figures. The simulations show regular fluctuations as we expect under *H_1_*. Although some of them go outside of the confidence interval, they are mainly embedded within it. The curves based on official results of Fig 10 show a similar behavior. Even the 2000 elections, which make a tour above the 2.58-level at moderate values of *k*, are well embedded within/in the confidence interval at large sample sizes. The standardized difference series from official results of Fig. 11 reach values higher than any simulation. They are well above the confidence interval, providing strong evidence against *H_1_* for elections of this group. Except for 2005, we firmly reject *H_1_* from 2004 onwards.

The alternative hypothesis to *H_1_* does not imply necessarily that there were fraudulent irregularities in the units with outlier values of *Z*; only that the extreme results occurred on a non-random set of electoral units. On this set, the vote counting has a significant bias in favor of Chávez. It is possible that there are non-fraudulent mechanisms that can explain this phenomenon. In fact, it is not unreasonable to think that some electoral districts have a greater chance of presenting non-fraudulent irregularities than others. But it is suspicious that it is only observed from 2004 onwards, with the sole exception of the 2005 parliamentary election. Inevitably, this points again to the 2004 Recall Referendum as a watershed regarding the integrity of the Venezuelan electoral processes.

### Detection of irregular variations in the electoral roll

Although we cannot discard that some of the anomalous patterns observed in elections since 2004 can be the result of non-fraudulent mechanisms that could affect the vote distribution, we have not found a convincing explanation of why the irregularities on the vote distribution were mainly observed in electoral units that favored Chávez. But, if there were election irregularities in those units, we have not estimated how they could affect the overall results. In light of the data collected over time, we tried a new approach to address this problem, which requires some preliminaries.

Electoral units in polling centers can be different from one election to the next. The number is determined by the electoral referee, who considers the number of registered voters in polling centers, among other control variables. Nevertheless, many polling centers are common in two consecutive elections. Thus, we will henceforth consider only results by polling center.

As mentioned earlier, one concern of the opposition is the possible fraudulent manipulation of the electoral register, which has grown dramatically over the last few years. In the twelve years between the presidential elections of 2000 and 2012, it increased by roughly 60% whereas the Venezuelan population grew significantly less between 2001 and 2011 (around 16% according to the projections of the Venezuelan census bureau). This is a controversial demographical problem *per se* but here we are only interested on how the growth is correlated with votes for Chávez.

Denote by *m*(*t*) the number of registered voters in a polling center in the electoral year *t*. Let us denote by 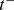 the most recent past election year. For example, if 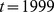 then 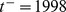 But, if 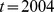 then 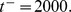 Consider now the *inter-annual growth* in the center at year *t*, defined by
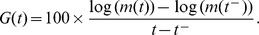



This indicator corresponds to the standard measure of inter-annual population growth. To get an idea of what the range of *G*(*t*) should be, the highest inter-annual population growth reported in Venezuela was 4%, in the 60 s. The current level is 1.5%. However, *G*(*t*) may take values 20 times larger. Another claim of the opposition is the unauthorized relocation of registered voters from one center to another. This irregularity may generate negative values of *G*(*t*) farther than the expected. We consider the absolute value |*G*(*t*)| as the measure of the inter-annual variation in the center for the election year *t*. Thus, irregular variation can involve irregular growth or irregular relocations. We point out that, for any year and any center, the inter-annual variation is measured in the same units.

**Figure 12:**
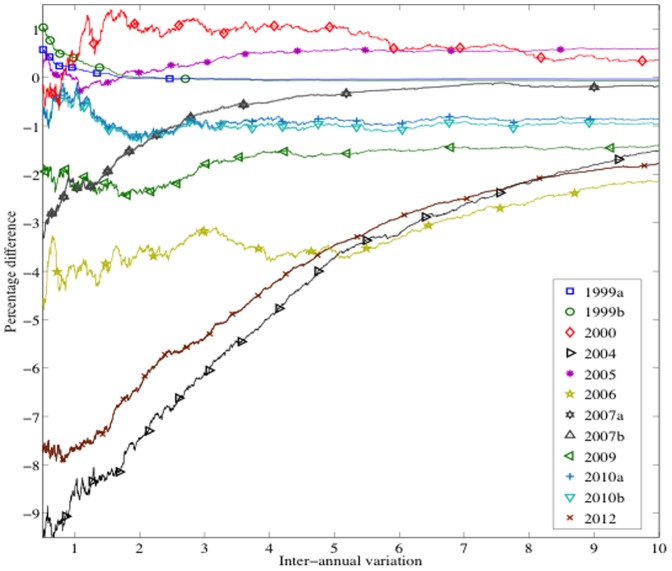
The correlation between irregular variations in centers and bias in the vote counting can be visualized by computing the proportion of votes favoring Chávez as a function of the inter-annual variation. The curves are centered, by subtracting the overall percentage of votes obtained by Chávez en each case.

**Figure 13:**
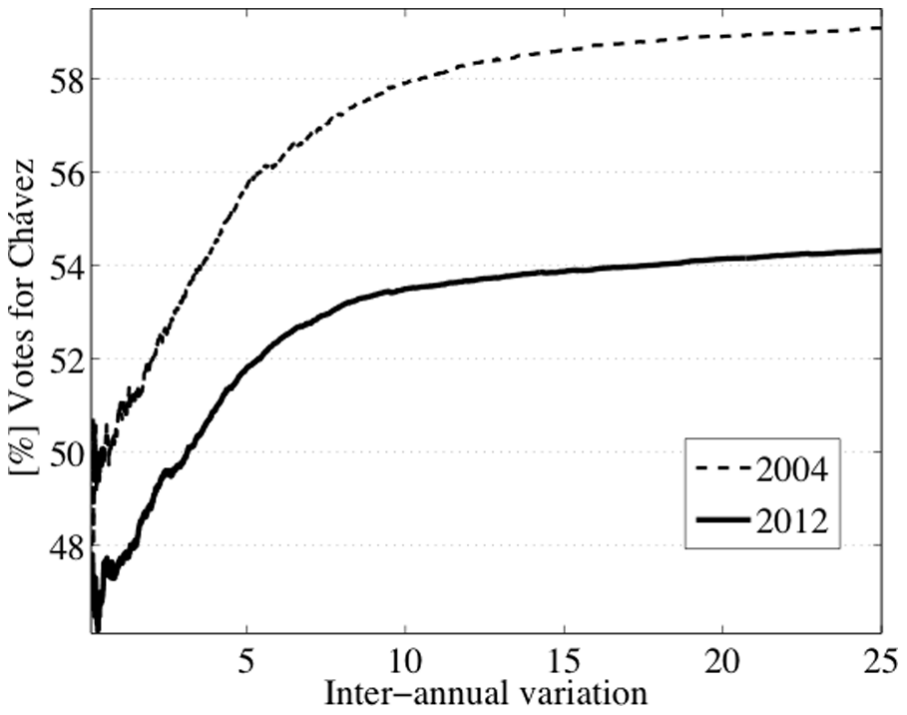
Percentage of votes favoring Chávez as a function of the inter-annual variation in the number of voters registered at polling centers. The official results of these elections are reached at extremely large values of the inter-annual variation. At small values, up to 69% of the total valid votes, the results are tight for the 2004 Recall Referendum. At moderate levels, up to the 79% of the total valid votes, the results are adverse for Chávez in the 2012 Presidential Elections.

A way to visualize the effect of irregular variations in centers is by computing the proportion of votes favoring Chávez as a function of |*G*(*t*)|: for each value |*G*(*t*)|, we compute the percentage of votes for Chávez (over valid votes) from centers with this value or lower. In order to compare the results for the different elections on the same scale, we centered the curves by subtracting the overall percentage of votes obtained by Chávez that year, denoted by *R* (Fig. 12). The curves previous to 2004 and the 2005 curve show a slight positive fluctuation. The rest of the curves follow a different pattern: a negative fluctuation (slight for 2010 and moderate for 2006 and 2009) or a nonlinear negative relationship (for 2004, 2007 and 2012). 2004 and 2012, where Chávez does not reach 50%, deserve special attention, if we exclude the centers with high inter-annual variations. We show the non-centered plots of these two cases on a more extended range of values of |*G*(*t*)| (Fig. 13). The plots show:

1. For the 2004 Recall Referendum, tight results in centers with variation of less than 1%. We note that these centers represent up to 69% of the total valid votes.
2. For the 2012 Presidential Election, adverse results in centers with variation of less than 4%. These centers represent up to 79% of total valid votes.
3. A strong increase close to extreme values of |*G*(*t*)|.

Until the 2000 Presidential Elections, the historical growth rate of the electoral roll was 11% every five years. The growth rate for the 2004 Recall Referendum was more than twice larger. This notable increment was a direct consequence of *misión identidad*: a Chávez program to provide ID cards, throughout the length and breadth of the country, which involved voter inclusion on the electoral roll. The new voters mostly came from excluded sectors: poor, rural and indigenous areas where the population had no access to many rights. According to conventional wisdom, they could have tenaciously supported Chávez [26]. If this were true, this would explain the increasing curve of the 2004 Recall Referendum. But even so, the growth of the curve should attenuate to the extent that the electoral register approached the voting age population, as in fact happened between 2006 and 2010. Hence, we cannot explain the increasing curve of 2012, when the coverage of the electoral register was approximately 97% and the growth rate, from 2010, just over 6%. Going back to 2004, there is evidence that the presidential elections of 1998 and 2000 were monotonic in class voting [27]. This means that the poor were most likely to vote for Chávez. But different survey data suggest that 2004 was not class voting monotonic. In fact they suggest that the support from poor people decreased from 60% at the time of the 2000 elections to about 30% for 2004, largely as a consequence of the abstention rates in this sector [28]. Chávez's success was the result of the electoral support from all socioeconomic classes [26]. The new constituents of 2004 did not vote mostly for Chávez as many people might think. Accordingly, we must discard the previous conjecture about why the curve of 2004 is increasing. Both, 2004 and 2012, show an irregular pattern that suggests a strategic inter-annual variation in the electoral roll. Furthermore, this variation was decisive for winning the 50% majority.

## Discussion and Results

Since the establishment of democracy in 1958, Venezuelan elections were considered free and fair until the 1993 Presidential Elections. Although the electoral outcome was accepted, and the winner did not face any legitimacy problem [29], the results of the 1993 election were questioned. This episode revealed a problem that worsened during the 90 s, the increasing mistrust in the results and the electoral system. There were frequent complaints of fraud in regional and local elections, and extreme partisanship on the part of the electoral referees. Partly to address these problems, a new electoral law was passed in 1997 [30]. The restoration of confidence was fundamental for the 1998 Presidential Elections in which, for the first time, Venezuelans were using an automated voting system. In addition, it is worth remembering the polarization of citizens and sociopolitical actors around the leadership and project of a populist outsider (former lieutenant Hugo Chávez, who had led a failed coup d'état in 1992) that challenged a sociopolitical model already in deep crisis. Despite some problems, elections were considered clean and transparent by both international observers and political parties [17]. Chávez won with 56.20% of the vote.

The 1999 and 2000 electoral processes were carried out with roughly the same voting system used in 1998 [31]. However, there were important differences between them. The referenda and elections of 1999 were characterized by low turnout and the use by Chavez of a strategic schema in order to maximize his electoral support, the so-called *kino* (a type of bingo card). After a year and a half of far-reaching sociopolitical transformations, including the promulgation of a new constitution, criticism towards Chavez's government increased in many sectors of society. In this context, as the 2000 elections approached, doubts were raised about the reliability of elections in great part because of the appointment of a biased CNE, disregarding constitutional procedures, and the changes introduced to the electoral system after the deadline set in the 1999 Constitution. Though the elections were flawed due to important organizational deficiencies and multiple complaints over irregularities, international observers validated the electoral results: Chávez was elected to his second term with 59.76% of valid votes [30], [31].

The recall referendum of 2004 has been widely analyzed [1], [10], [21], [32]–[35]. Unlike the rest of the case studies that we have considered, only 150 electoral units were audited, and it has been demonstrated that the sample was neither representative nor random [32]. Additionally, there is evidence that the vote count could have been altered for a high percentage of automated electoral units in the processing center [33]. Moreover, in this year the electoral register experienced a crucial inter-annual growth (around 9%). The possibility of the election being rigged generated a deep lack of confidence in the electoral system, which led the opposition to not participate in the 2005 legislative elections.

From 2006, the CNE introduced important improvements. These included better infrastructure, more guarantees for the secrecy of the ballot, and an increase of the audits carried out on the system. In particular, the post-election audits now involve more than 54% of the electoral units. During this period, the electoral council also continued with the campaign initiated in 2004, aimed at the inclusion of new voters. The improvements have led to a growing confidence in elections as a tool for political change [36]. Despite this progress, there still exist major concerns on issues such as the hegemonic manipulation of the electoral registry (including unexpected reallocation of voters and massive inclusions of new voters after voter registration deadlines), and implementation of technological platforms (voting machines and fingerprint scans) that raise distrust among the electorate. To this we should add the use and abuse of public resources by the government for the electoral campaigns. All these irregularities have been reported to international and national observers and civil organizations [37]–[40]. Although the elections took place on an uneven playing field, in detriment of the opposition, the Chávez victories in the 2006 Presidential Elections and in the 2009 referendum are not questioned partly because post-election audits do not disagree from overall results significantly. It was a different story in 2007. The referendum on the constitutional reform was Chávez's first defeat in nationwide elections. The opposition officially won by a narrow margin (less than 1% of the votes), but the definitive results, including the behavior of approximately 11% of the electoral census, remains unknown. Unlike in other elections, the audits did reflect important differences between the votes cast and those audited. A quick count based on a sample of audited electoral units estimated a difference greater than 8% [37]. It pointed towards the direction of a possible manipulation of the vote count. The possibility that the CNE had tried to make up the results with little room for maneuver may not be ruled out. The large mobilization of wide sectors of the opposition and their presence at the voting centers would have made it difficult to overturn the results.

The 2010 Parliamentary Elections were preceded by a new electoral reform. Under the approved system, the percentage of deputies of the National Assembly elected by nominal election increased from 60% to 70%. Furthermore, the reform legalized a practice with which the government's party had been clearly overrepresented since the 2004 regional elections (colloquially called as *morochas*). In addition, there were modifications in the electoral districts which indicated gerrymandering as was rendered evident by the double strategy employed: isolating and/or concentrating zones that had voted against Chávez and his supporters in the past, and uniting areas with an electoral behavior favorable to the Government with others that historically opposed him. In the end, the government party, The United Socialist Party of Venezuela (PSUV in Spanish), and its allies, had the most votes, but they obtained less than 50% of valid votes. Nevertheless, the changes clearly favored the PSUV in the National Assembly (it obtained 98 seats out of 165, one of the seats corresponds to one of his allies). The opposition criticized the reform but accepted the results of the vote count [41].

With the above discussion, we wish to emphasize that irregularities and problems were common in all electoral processes during the Chávez period. In elections previous to the recall referendum, these irregularities were not enough to question the results, which were accepted by the main political actors. Otherwise, the nature and range of the irregularities after 2004 have deeply concerned the opposition, but in the end the results were also accepted for practical reasons. We must remember that the opposition denounced fraud in 2004 and with this decision it initiated a costly political strategy for two years, in particular renouncing a fundamental space in the parliament elected in 2005. Our election forensics is consistent with this analysis:

- We detected no signs of fraud for elections and referenda previous to 2004.
- We found anomalous statistical patterns, which may be traces of election irregularities, in electoral processes between 2006 and 2010.
- We cannot discard outcome-determinative fraud in the 2004 referendum, as has been already reported [10], [21], [32]–[34].

Our analysis for the 2012 presidential elections offers a controversial finding. We find statistical evidence, which may be interpreted as signals of systematic election irregularities, similar to that observed for 2004. Contrary to the opinion of radical sectors, which did not accept the results of the elections, the opposition candidate conceded defeat [42]. The opposition forces thus avoided returning to the strategy pursued in the past. Possibly, they weighed having no evidence of massive fraud and the call for new presidential elections in a short time due to the illness of the president (he survived only five months longer). However, these elections raise at least three questions.

Firstly, both international observers and the opposition have recommended for years a full audit of the electoral register. This has undergone tremendous changes with the voter inclusion program (*misión identidad*) started before the 2004 referendum. According to official figures (http://www.ine.gov.ve), the coverage of the electoral register went from approximately 75% in 2000 to 97% in 2012, involving a growth of 60% in the number of voters. The consistency of these changes is controversial. Some scholars think that they are out of proportion [43] while others do not [44]. International standards accept a certain level of inaccuracies as long as no partisan bias in favor of or against a political party is detected [45]. We have argued that the growth of the register is strongly correlated with the *Chavista* vote, particularly in 2004 and 2012.

Secondly, while the opposition made a great effort to be present at the post-election audits, they failed to meet their goal. The most basic element in the post-election audits is the manual verification of the number of total votes in all the electoral units. This simple procedure was taken in only approximately 6% of the electoral units [46]. Therefore, there are no guarantees that the register is not inflated, as many people believe [45]. Consequently, the reliability of the 2012 post-election audit is subject to question. In fact, the systematic statistical irregularities discussed in this paper suggest strategic anomalous variations in the register that favor Chávez in the vote count.

Thirdly, these elections were marked by a large number of electoral complaints [47] that have been increasing since 2007 [48], when Chávez radicalized his political project [49]. Even though it is impossible to quantify the impact of the denounced anomalies they certainly discredit the overall results, in line with the outputs of the statistical methods that we have used.

## Conclusions

In this paper, we have applied four different forensic analyses for the Venezuelan national elections held during the Chávez mandate. In particular, we discussed the use of the second-digit Benford's law, two different approaches for the statistical detection of systematic election irregularities, and a tool based on the evolution of the electoral register. In order to reach a better understanding of the obtained results, we have placed them in their political context. Thus, we provide a thorough evaluation of the integrity of the electoral processes under study. Our results subscribe with the results of previous studies on the referenda of 2004 [10], [21], 2007 and 2009 [20] and shed new light on other elections, especially on the 2012 presidential elections.

In sum, we have found anomalous statistical patterns consistent with a hypothetical electoral fraud in the 2004 recall referendum and all elections and referenda held between 2006 and 2012. Although this does not mean that we provide concluding evidence of fraud, specifically of outcome-determinative fraud, this raises serious doubts regarding the impartiality of the current electoral authority and supports the allegations of fraud claimed by important sectors of the Venezuelan society. Our study calls into question the reliability of the electoral register, a major concern since 2004. In particular, we detected irregular variations in the electoral roll that could have overturned the results for the 2004 referendum and the 2012 elections. As a corollary to our analysis, we recommend monitoring polling centers where atypical support (extreme *Z*-values) occurs systematically. We also strongly recommend a full audit of the register. Without it, there is no certainty of the validity of post-election audits. These are considered the main guarantor of an electronic voting system, recently recognized by former president Jimmy Carter as “the best in the world” [50]. Taking into account the multiple irregularities in the Venezuelan vote, which are difficult to quantify even if they are detected by different election forensic tools, and the possible strategic growth of the register to favor Chávez that we have discussed, we think Mr. Carter may be wrong.
